# Supplementary material for: Deep learning modeling m6A deposition reveals the importance of downstream cis-element sequences
Source: Nat Commun. 2022 May 17;13:2720. doi: 10.1038/s41467-022-30209-7 (PMC9114009; doi:10.1038/s41467-022-30209-7)
Supplement: Supplementary file 1 — Supplementary Information [file 41467_2022_30209_MOESM1_ESM.pdf]

Supplementary Fig.1: iM6A models m^6A deposition with single nucleotide resolution in human genome

a, Receiver operator curves (ROCs) and corresponding area under receiver operator curves (AUROC) scores of iM6A, CNN-RNN, and SVM. Here human chromosome 9 data was used to test the iM6A, CNN-RNN and SVM models, which were trained independently on data of other human chromosomes except chromosome 9.

b-c, Precision-Recall curves (PRCs) and corresponding area under precision-recall curves (AUPRC) scores of iM6A, CNN-RNN, and SVM. Here chromosome 9 data was used to test the iM6A, CNN-RNN and SVM models, which were trained independently on data of other chromosomes except chromosome 9.

d, Heatmap of the iM6A modeling and m^6A-CLIP detected sites in human chromosome 9. The modeled sites were sorted based on modeled score, the black line denoted whether methylation was identified at the sites by m^6A-CLIP.

e-k, Heatmap of the iM6A modeling and various experimentally determined m^6A sites, including MAZTER-seq (conformed to RRACA motif), m^6A-label-seq, m6ACE-seq, and miCLIP2. The modeled sites were sorted based on modeled score, the black line denoted whether methylation was identified at the sites. The experimental method and specie were labeled as figure showed.

l, Number of m^6A sites detected by different methods including m^6A-CLIP, m^6A-label-seq, m6ACE-seq and MAZTER-seq in mouse and human.

m, The modeled probability by iM6A agreed with the experimentally quantified m^6A methylation level. Modeled probability by iM6A (left panel) and enrichment score quantified by m^6A-CLIP (right panel) at human m^6A sites. The m^6A sites were categorized into three groups based on their m^6A peak enrichment value as the low (n=468924), medium (n=73217), and high (n=2035) groups. Median and interquartile ranges are presented for the box plot.

n-o, The modeled probability by iM6A agreed with the experimentally quantified m^6A methylation level. Modeled probability by iM6A (left panel) and cleavage

efficiencies quantified by MAZTER-seq (which correlated negatively with m^6A methylation level, right panel) at mouse m^6A sites (Supplementary Fig. 1n) or human m^6A sites (Supplementary Fig. 1o). The m6A sites were categorized into five groups as the control (n=33749 for mouse, n=45508 for human), low (n=542 for mouse, n=2470 for human), medium (n=615 for mouse, n=908 for human), high (n=681 for mouse, n=378 for human), and highest (n=95 for mouse, n=162 for human) groups. Median and interquartile ranges are presented for the box plot.

p, Scatter plot of modeled probability for m^6A sites (n=100000) in human chromosome 9 using human RAC iM6A model versus human RRACH iM6A model. Both models were trained independently on data of other human chromosomes except chromosome 9. Each dot represented one site in human chromosome 9 discovered by both models, and the labelled axes provided the probability values for that site by the two models.

q, Receiver operator curves (ROCs) and corresponding area under receiver operator curves (AUROC) scores of iM6A with different sequence length (80, 400, 2000, and 10000 nt). Here mouse chromosome 9 data was used to test the iM6A, which were trained independently on data of other mouse chromosomes except chromosome 9.

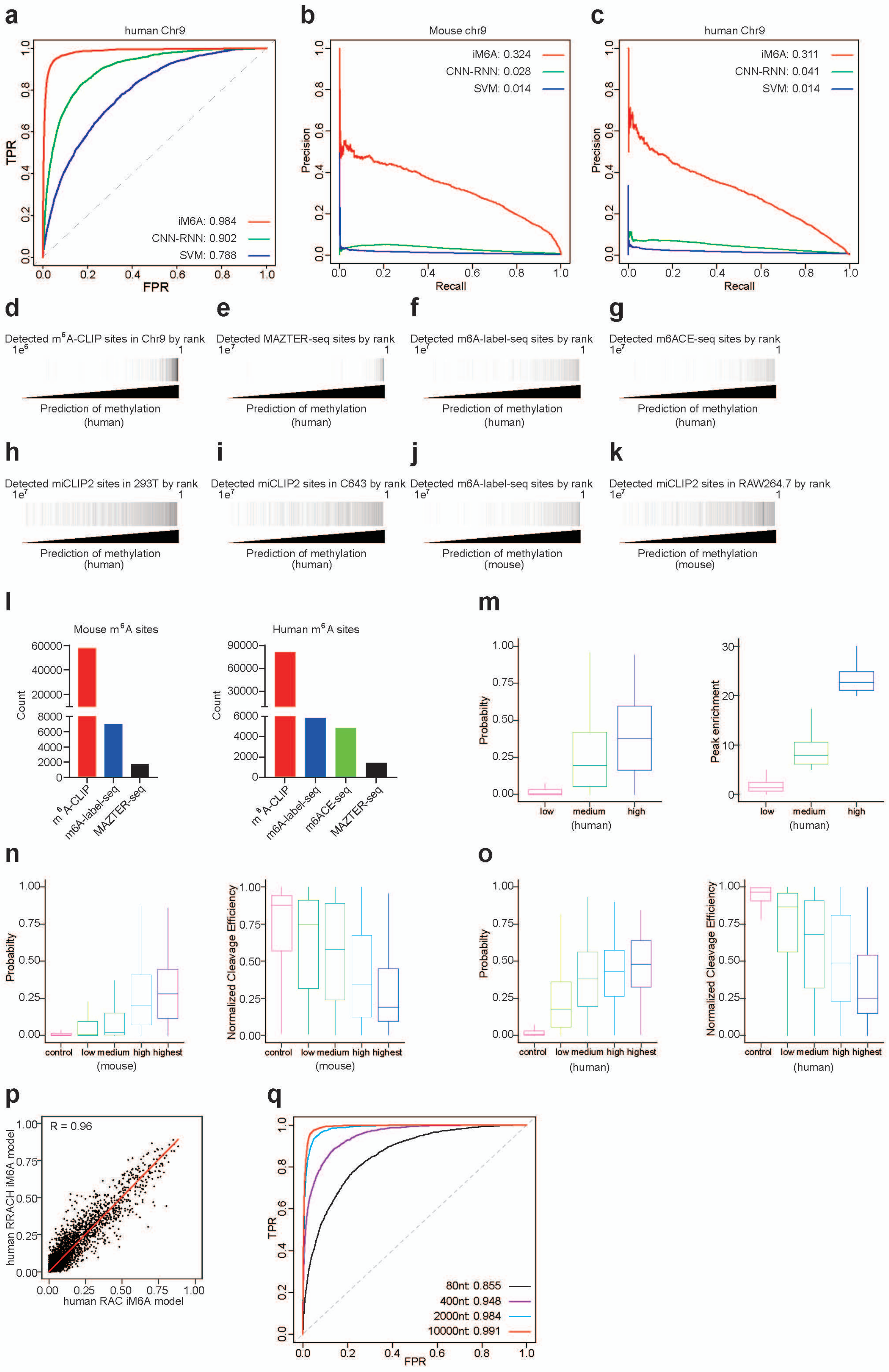
a
human Chr9
TPR
FPR
iM6A: 0.984
CNN-RNN: 0.902
SVM: 0.788
b
Mouse chr9
Precision
Recall
iM6A: 0.324
CNN-RNN: 0.028
SVM: 0.014
c
human Chr9
Precision
Recall
iM6A: 0.311
CNN-RNN: 0.041
SVM: 0.014
d
Detected m6A-CLIP sites in Chr9 by rank
Prediction of methylation (human)
e
Detected MAZTER-seq sites by rank
Prediction of methylation (human)
f
Detected m6A-label-seq sites by rank
Prediction of methylation (human)
g
Detected m6ACE-seq sites by rank
Prediction of methylation (human)
h
Detected miCLIP2 sites in 293T by rank
Prediction of methylation (human)
i
Detected miCLIP2 sites in C643 by rank
Prediction of methylation (human)
j
Detected m6A-label-seq sites by rank
Prediction of methylation (mouse)
k
Detected miCLIP2 sites in RAW264.7 by rank
Prediction of methylation (mouse)
l
Mouse m6A sites
Count
m6A-CLIP
m6A-label-seq
MAZTER-seq
Human m6A sites
Count
m6A-CLIP
m6A-label-seq
m6ACE-seq
MAZTER-seq
m
Probability
low
medium
high
(human)
Peak enrichment
low
medium
high
(human)
n
Probability
control
low
medium
high
highest
(mouse)
Normalized Cleavage Efficiency
control
low
medium
high
highest
(mouse)
o
Probability
control
low
medium
high
highest
(human)
Normalized Cleavage Efficiency
control
low
medium
high
highest
(human)
p
R = 0.96
human RRACH iM6A model
human RAC iM6A model
q
TPR
FPR
80nt 0.855
400nt 0.948
2000nt 0.984
10000nt 0.991

Supplementary Fig.2: Cis-elements that regulate m^6A deposition locate largely within 50nt downstream of m^6A sites

a-b, Positional plot of ΔProbability (cutoff = 0.1) for the human m^6A sites located in last exon (Supplementary Fig. 2a) or long internal exon (Supplementary Fig. 2b). Up panel: dot plot of ΔProbability for the sequences (-250 to 250) around the m^6A site. Bottom panel: dot plot of ΔProbability for the sequences (-50 to 50) around the m^6A site. Red color dots were mutational events that increased m^6A probability; Green color dots were mutational events that decreased m^6A probability.

c-d, Dendrogram showed clustering of Top 20 enhancer motifs (Supplementary Fig. 2c) or Top 20 silencer motifs (Supplementary Fig. 2d) found in human last exon. The enhancers mostly contained part of RRACH motif, the silencers mostly contained CG/GT/CT motifs.

e, Scatter plot for the effect correlation for all pentamers between the study in human last exon and the study in human long internal exon. The effect of each pentamer motif was determined by the slope of linear regression equation, and each grey dot was a pentamer.

f-g, Dendrogram showed clustering of Top 20 enhancer motifs (Supplementary Fig. 2f) or Top 20 silencer motifs (Supplementary Fig. 2g) found in mouse long internal exon. The enhancers mostly contained part of RRACH motif, the silencers mostly contained CG/GT/CT motifs.

h-i, Scatter plot for the effect correlation for all pentamers between the study in last exon (Supplementary Fig. 2h) or long internal exon (Supplementary Fig. 2i) of mouse and human. The effect of each pentamer motif was determined by the slope of linear regression equation, and each grey dot was a pentamer.

j-k, Positional plot for the frequency of Top 100 enhancers (Supplementary Fig. 2j) or silencers (Supplementary Fig. 2k) in the sequences around the human m^6A sites. The plots were compared between higher m^6A probability (red color, probability >= 0.7) and lower m^6A probability (control, green color, probability < 0.1) of RAC sites. Data were presented as mean ± S.E.M. (standard error of

the mean). (Using other top number of enhancers or silencers generate similar results)

l, Positional plot for conservation score in the sequences around the human m^6A sites. The plots were compared between higher m^6A probability (red color, probability >= 0.7) and lower m^6A probability (control, green color, probability < 0.1) of RAC sites. Data were presented as mean ± S.E.M. (standard error of the mean).

m-n, Positional plot for the frequency of RAC sites in the sequences around the m^6A sites (Supplementary Fig. 2m) or non-m^6A sites (Supplementary Fig. 2n). The plots were compared between the methylated RAC sites (red color, higher m^6A probability, probability >= 0.05) and the non-methylated RAC sites (green color, lower m^6A probability, probability < 0.05). Data were presented as mean ± S.E.M. (standard error of the mean).

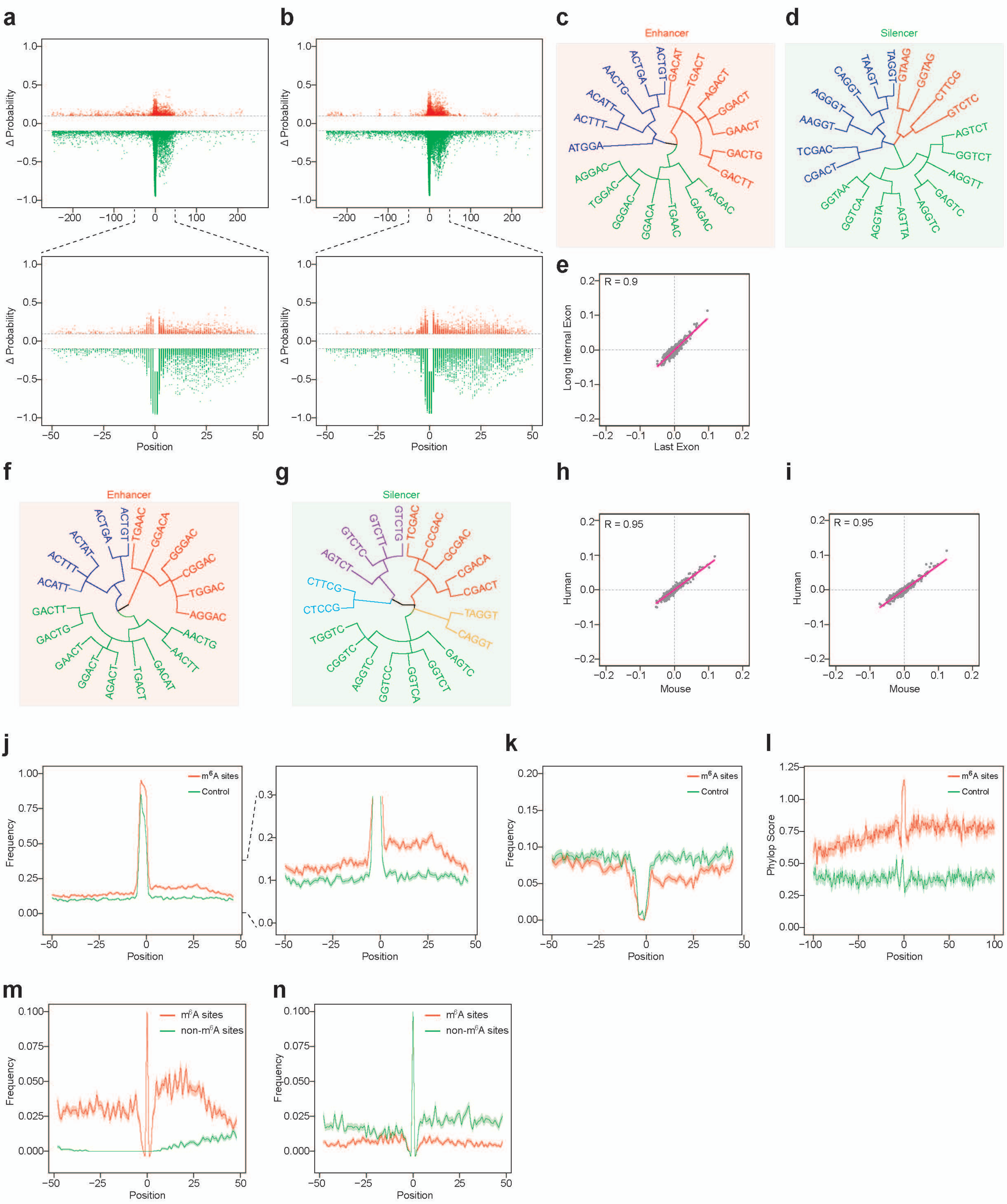

a
b
c
d
Enhancer
Silencer
Δ Probability
Position
e
R = 0.9
Long Internal Exon
Last Exon
f
g
h
i
R = 0.95
Human
Mouse
j
k
l
m
n
m6A sites
Control
non-m6A sites
Frequency
Phylop Score

Supplementary Fig.3 The experimentally determined m^{6}A sites by different experimental approaches have similar m^{6}A enhancers and silencers positional distributions.

a-n, Positional plot for the frequency of Top 100 enhancers (Supplementary Fig. 3a,c,e,g,i,k,m) or silencers (Supplementary Fig. 3b,d,f,h,j,l,n) in the sequences around the experimentally determined m^{6}A sites. The plots were compared between m^{6}A-CLIP sites (red) and the exact RAC motif matched non-m^{6}A sites as control (green). Data were presented as mean ± S.E.M. (standard error of the mean). (Using other top number of enhancers or silencers generate similar results). The experimental methods and species were labeled in each panel.

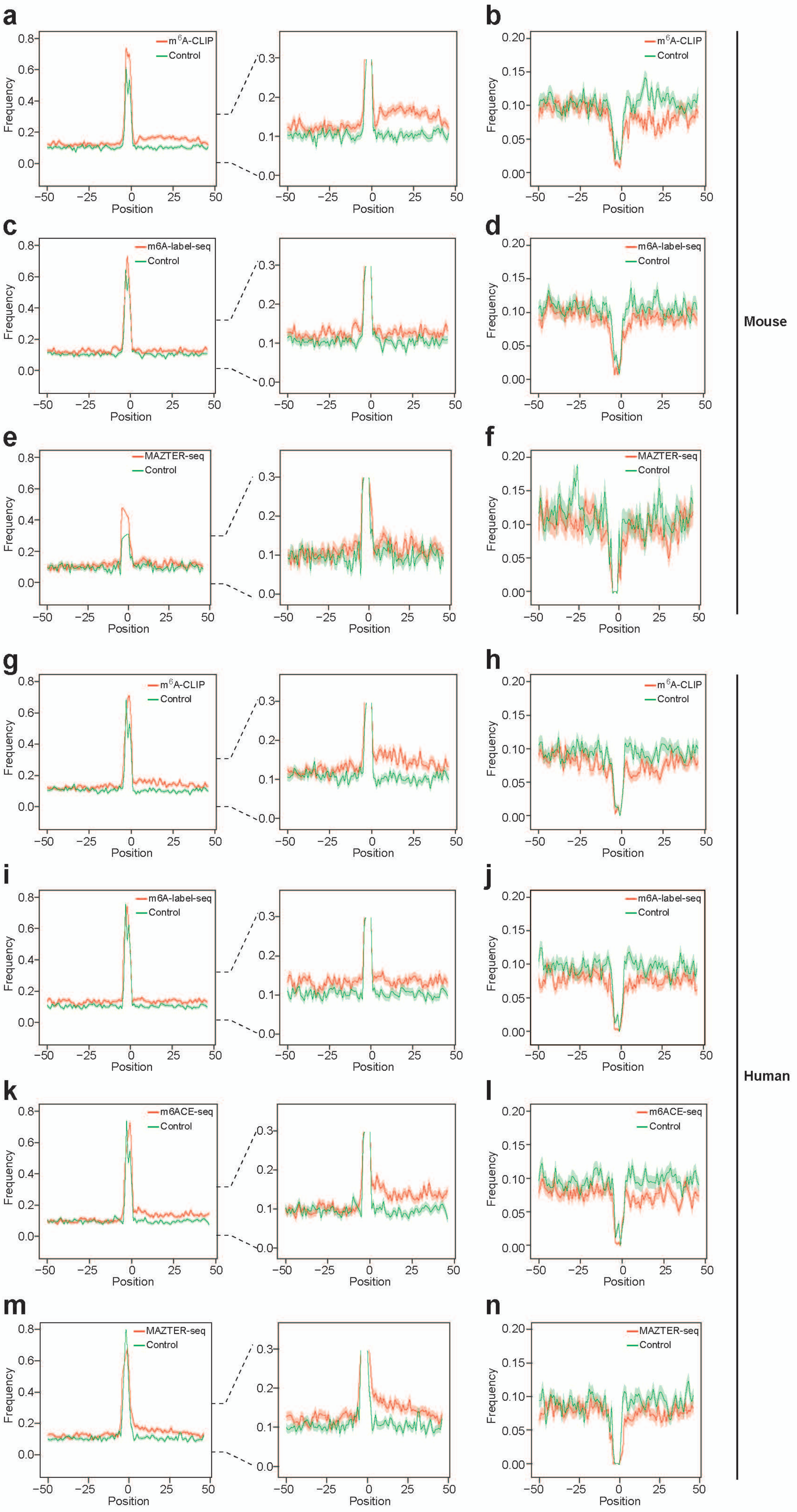

a
b
c
d
e
f
g
h
i
j
k
l
m
n
m6A-CLIP
m6A-label-seq
MAZTER-seq
m6ACE-seq
Control
Frequency
Position
Mouse
Human

Supplementary Fig.4: Synonymous mutation of SNVs influence m^6A deposition

a, Scatter plot of predicted probability for m^6A sites with major allele (ProbREF) or minor allele (ProbALT), all SNVs are synonymous mutations. Red color dots were mutational events that increased m^6A probability (ΔProbability>=0.1); Green color dots are mutational events that decreased m^6A probability (ΔProbability <= -0.1).

b, Positional plot of ΔProbability (cutoff = 0.1) for m^6A sites with major allele or minor allele, all SNVs are synonymous mutations. Red color dots were mutational events that increased m^6A probability; Green color dots were mutational events that decreased m^6A probability.

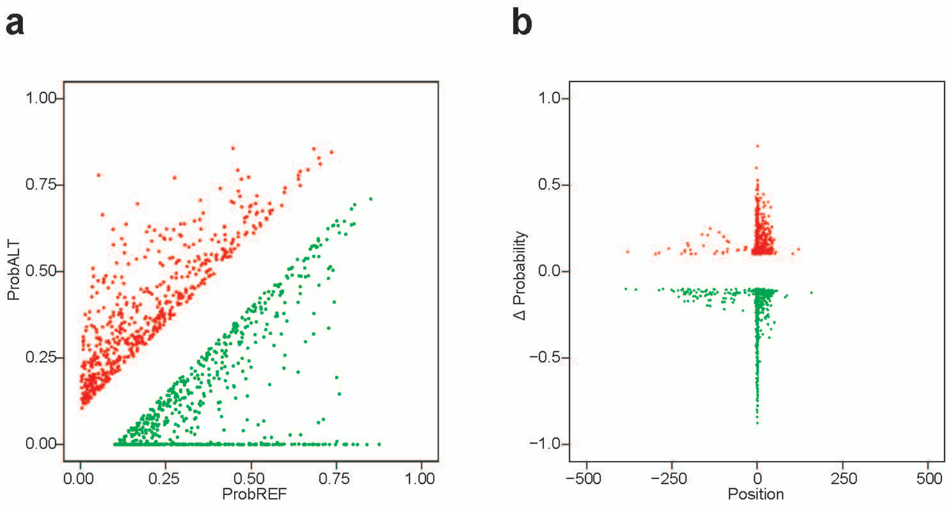
a
b
1.00
0.75
0.50
0.25
0.00
ProbALT
0.00
0.25
0.50
0.75
1.00
ProbREF
1.0
0.5
0.0
−0.5
−1.0
Δ Probability
−500
−250
0
250
500
Position

Supplementary Fig.5: Synonymous codons may influence m^6A deposition

a, Positional plot of ΔProbability (cutoff = 0.1) for saturation synonymous codon swap. Red color dots were those codon swap events that increased m^6A probability; Green color dots were those codon swap events that decreased m^6A probability.

b, Bar plot of effect values for top 10 enhancer codons and top 10 silencer codons, its corresponding amino acids were also labeled

c-d, Positional plot for the frequency of Top 20 enhancer codons (Supplementary Fig. 5c) or silencer codons (Supplementary Fig. 5d) in the sequences around the m^6A sites. The plots were compared between higher m^6A probability sites (red color, probability >= 0.7) and lower m^6A probability sites (the exact RAC motif-matched control, green color, probability < 0.01). Data were presented as mean ± SEM. (Using other top number of enhancer or silencer codons generated similar results)

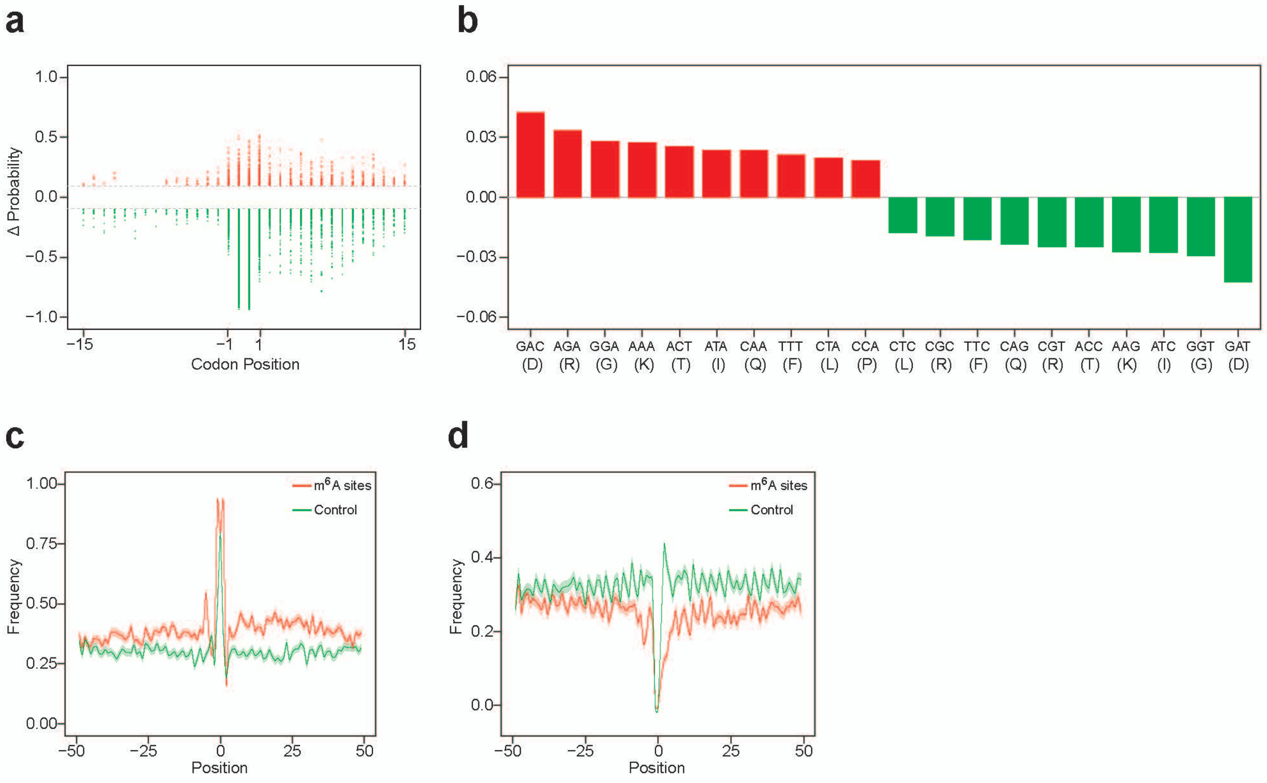
a
1.0
0.5
0.0
−0.5
−1.0
Δ Probability
−15
−1
1
15
Codon Position
b
0.06
0.03
0.00
−0.03
−0.06
GAC (D)
AGA (R)
GGA (G)
AAA (K)
ACT (T)
ATA (I)
CAA (Q)
TTT (F)
CTA (L)
CCA (P)
CTC (L)
CGC (R)
TTC (F)
CAG (Q)
CGT (R)
ACC (T)
AAG (K)
ATC (I)
GGT (G)
GAT (D)
c
1.00
0.75
0.50
0.25
0.00
Frequency
m6A sites
Control
−50
−25
0
25
50
Position
d
0.6
0.4
0.2
0.0
Frequency
m6A sites
Control
−50
−25
0
25
50
Position

Supplementary Fig.6: Stop codon TGA may favor m^{6}A deposition at and adjacent to Stop codon (human data).

a, Positional plot of average modeled m^{6}A probability around the stop codon, the position 0 was the T nucleotide for the stop codons. The red, green, and blue lines represented genes with TAA, TAG, or TGA as its stop codon respectively. Up panel: regions 500 nt upstream and downstream from 0 position. Bottom panel: regions 10 nt upstream and downstream from 0 position.

b, Positional plot for stop codon swap. First panel: illustrator of stop codon swap. Second panel: positional plot for TGA to TAA or TAG. Third panel: positional plot for TAA to TGA. Fourth panel: positional plot for TAG to TGA.

c, Cumulative distribution function (CDF) plot of modeled probability for TGA to TAA or TAG. The p-values were calculated by the Kolmogorov-Smirnov test (KS-test). (the left, middle and right panel for NRACTRR, TRACN and TRRACN motifs).

d, Cumulative distribution function (CDF) plot of modeled probability for TAA or TAG to TGA. The p-values were calculated by the Kolmogorov-Smirnov test (KS-test). (the left, middle and right panel for NRACTRR, TRACN and TRRACN motifs).

e, The m^{6}A sites were categorized into two groups (m^{6}A or non-m^{6}A) based on its probability value (the cutoff = 0.05), donut plot of percentage of stop codon for m^{6}A sites and non-m^{6}A sites (Left panel). Bar plot of $\log_2$(odd ratio, m^{6}A sites over non-m^{6}A sites) of percentage of stop codon (Right panel). The p-value was calculated by the Fisher's exact test.

f, Box plot of conservation score of stop codons with or without m^{6}A sites (n=1312 for TGA with m^{6}A sites, n=1304 for TGA without m^{6}A sites, n=787 for non-TGA with m^{6}A sites, n=950 for TGA without m^{6}A sites). The p-values were calculated by the one-sided Student's t-test (Significance: *** $P < 0.001$). Median and interquartile ranges are presented for the box plot.

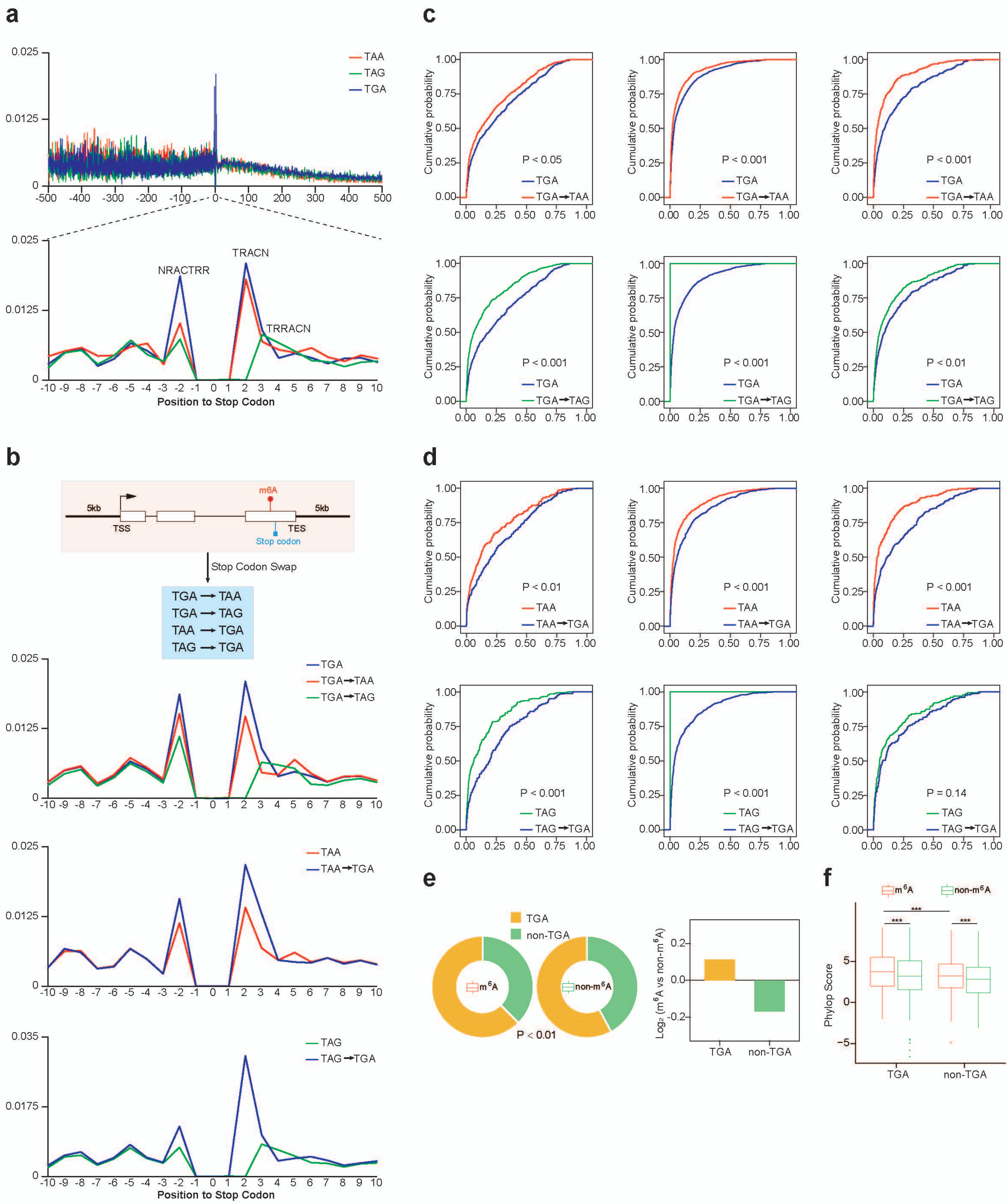

a
TAA
TAG
TGA
NRACTRR
TRACN
TRRACN
Position to Stop Codon
b
m6A
5kb
TSS
TES
Stop codon
Stop Codon Swap
TGA → TAA
TGA → TAG
TAA → TGA
TAG → TGA
TGA
TGA→TAA
TGA→TAG
TAA
TAA→TGA
TAG
TAG→TGA
Position to Stop Codon
c
Cumulative probability
P < 0.05
P < 0.001
P < 0.001
P < 0.001
P < 0.001
P < 0.01
d
P < 0.01
P < 0.001
P < 0.001
P < 0.001
P < 0.001
P = 0.14
e
TGA
non-TGA
m6A
non-m6A
P < 0.01
Log2 (m6A vs non-m6A)
f
m6A
non-m6A
Phylop Score
***
TGA
non-TGA

Supplementary Fig.7: Stop codon TGA is more conserved when it's part of m^{6}A site

a, Box plot of conservation score of stop codons with or without mouse m^{6}A-CLIP sites (n=306 for TGA with CLIP sites, n=291 for TGA without CLIP sites, n=166 for non-TGA with CLIP sites, n=187 for TGA without CLIP sites). The p-values were calculated by the one-sided Student's t-test (Significance: ** P < 0.01, *** P < 0.001). Median and interquartile ranges are presented for the box plot.

b, Box plot of conservation score of stop codons with or without human m^{6}A-CLIP sites (n=353 for TGA with CLIP sites, n=361 for TGA without CLIP sites, n=202 for non-TGA with CLIP sites, n=274 for TGA without CLIP sites). The p-values were calculated by the one-sided Student's t-test (Significance: ** P < 0.01, *** P < 0.001). Median and interquartile ranges are presented for the box plot

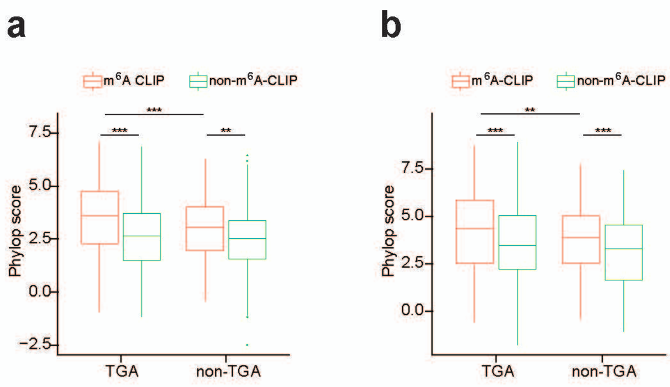
a
m6A CLIP
non-m6A-CLIP
***
***
**
Phylop score
7.5
5.0
2.5
0.0
−2.5
TGA
non-TGA
b
m6A-CLIP
non-m6A-CLIP
**
***
***
Phylop score
7.5
5.0
2.5
0.0
TGA
non-TGA

Supplementary Table 1 Number of motifs containing TGA, TAA, TAG in m6A enhancer motifs

		Top50	Top100	Top150
Enhancer	TGA	5	13	21
	TAA	0	3	13
	TAG	0	0	0
