# Supplementary material for: Deep learning modeling m6A deposition reveals the importance of downstream cis-element sequences
Source: Nat Commun. 2022 May 17;13:2720. doi: 10.1038/s41467-022-30209-7 (PMC9114009; doi:10.1038/s41467-022-30209-7)
Supplement: Supplementary file 3 — Description of Additional Supplementary Files [file 41467_2022_30209_MOESM3_ESM.pdf]

Description of Additional Supplementary Files

File Name: Supplementary Data 1
Description: Hyperparameter search of iM6A. Different model structures (6 in total, the Model structure table) and batch sizes (6 in total, the Batch size table)

File Name: Supplementary Data 2
Description: Effect values of pentamer motifs. Four situations are provided (human long internal exon, human last exon, mouse long internal exon and mouse last exon). The p value is calculated by student t test in linear regression.

File Name: Supplementary Data 3
Description: Effect values of codons. Both human and mouse last exon codon effect values are provided. The p value is calculated by student t test in linear regression.
